# Isolated cutaneous acanthamoebiasis under prophylactic anticryptococcal treatment in an immunocompromised patient

**DOI:** 10.1016/j.jdcr.2022.08.008

**Published:** 2022-08-10

**Authors:** Samuel A. Stetkevich, Stephanie T. Le, Adam R. Ford, Alain Brassard, Maija Kiuru, Maxwell A. Fung, Danielle M. Tartar

**Affiliations:** a Division of Dermatology, Department of Medicine, University of Toledo College of Medicine, Toledo, Ohio; b Department of Dermatology, University of California, Davis, Davis, California; c Department of Dermatopathology, University of California, Davis, Davis, California

**Keywords:** acanthamoeba, combined variable immune deficiency (CVID), cryptococcus, CNS, central nervous system

## Introduction

Acanthamoeba species are unicellular, microscopic protozoans that live ubiquitously and cause severe, and sometimes fatal, infections in immunocompromised patients. Infections are classically described in the eye or central nervous system (CNS). Extracerebral acanthamoebiasis, including skin manifestations, may occur in the setting of disseminated disease. Herein, we provide a rare case of cutaneous acanthamoebiasis lacking CNS and eye involvement and in the setting of long-term antifungal therapy for a concomitant disseminated cryptococcal infection.

## Case presentation

An 81-year-old female who was ultimately diagnosed with combined variable immunodeficiency (CVID) and had a history of *Mycobacterium tuberculosis* and *Mycobacterium chelonae* infections was admitted for worsening headaches and epigastric pain. One year prior to presentation, she had been diagnosed with disseminated cryptococcal infection, including both meningitis and pneumonitis. At that time, she underwent induction therapy with intravenous liposomal amphotericin B 200 mg once daily and oral flucytosine 1000 mg twice daily for a total of 17 days. She was then transitioned to chronic management with oral fluconazole 100 mg daily, which she was on at the time of the current presentation. The patient was immediately started on repeat induction therapy with intravenous liposomal amphotericin B 200 mg once daily and oral flucytosine 1000 mg twice daily due to concern for relapsing cryptococcal meningitis.

**Fig 1:**
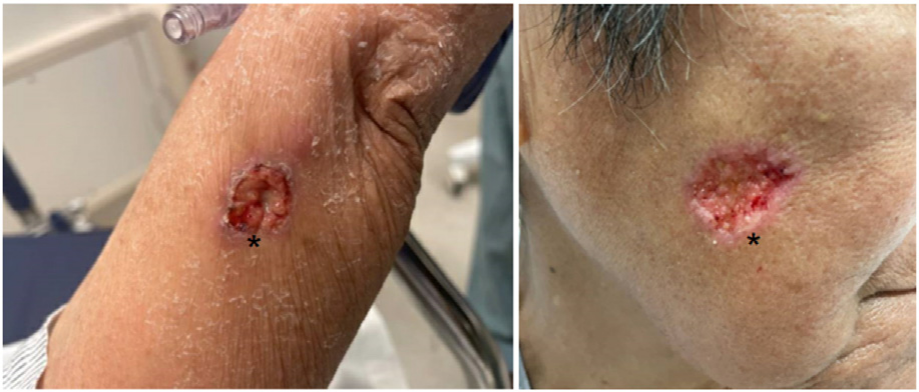
Acanthamoeba, well-demarcated ulcerations with prominent granulation tissue on her left upper arm and right cheek (*asterisks*).

**Fig 2:**
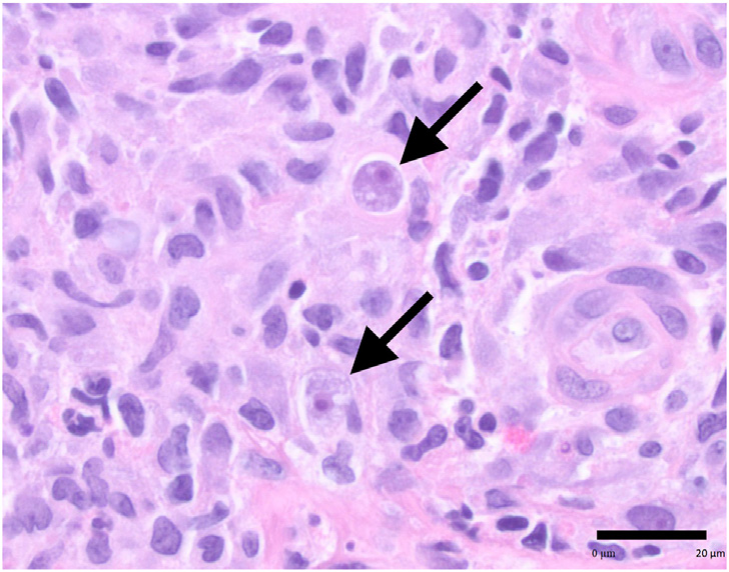
Amebae (*arrows*) with vacuolated cytoplasm and a single “fish eye” nucleus. *Scale bar* = 20 μm (hematoxylin-eosin stain).

She was additionally found to have tender, enlarging well-demarcated ulcerations with prominent granulation tissue on her right cheek and left upper arm. These had been present for 14 m and had previously been interpreted as cryptococcus but were not responding to therapy leading to dermatology consultation (Fig 1). Two punch biopsies were performed from the edge of the left upper arm lesion, one for hematoxylin and eosin staining and one for tissue culture. Histopathology revealed suppurative granulomatous dermatitis concerning for infection. Periodic acid–Schiff plus distase, periodic acid–Schiff, Grocott methenamine silver, Fite, Fontana-Masson, and gram stains were negative for cryptococcus, other fungal pathogens, gram-positive bacteria, and mycobacteria. Viral swab of the lesions was negative for herpes simplex virus/varicella-zoster virus. Serologies were negative for acute or recent infection of Epstein-Barr virus ((+)EB NA IgG, (-)EB EA IgG, (+)EBV VCA IgG, (-)EBV VCA IgM) and cytomegalovirus. Bacterial, fungal, and acid-fast cultures were all negative. Given the negative infectious workup but continued concern for infectious etiology, an additional punch biopsy was performed on the left upper arm and sent for broad-range polymerase chain reaction and next-generation sequencing at the University of Washington.^1^ Acanthamoeba species was identified, and rare organisms with vacuolated cytoplasm and a single “fish eye” nucleus characteristic of acanthamoebiasis were identified upon review of the initial histopathologic slides (Fig 2). Acanthamoeba infection was confirmed by the Centers for Disease Control and Prevention after histopathological review.

**Fig 3:**
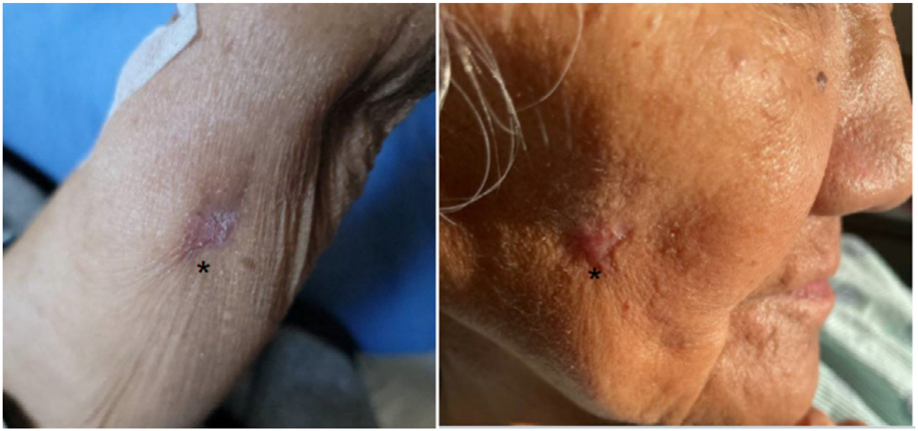
Acanthamoeba, re-epithelization of prior ulcerations following escalated therapy (*asterisks*).

Intravenous liposomal amphotericin B was discontinued and oral flucytosine was increased to 1500 mg daily. High-dose oral fluconazole 600 mg daily, intravenous pentamidine 165 mg daily, oral miltefosine 50 mg twice daily, and oral sulfadiazine 1500 mg every 6 hours were also added to the treatment regimen per infectious disease. Repeat lumbar puncture and brain magnetic resonance imaging did not show evidence of CNS disease; therefore, pentamidine was discontinued within 1 week. Miltefosine was continued for 6 weeks, until the cutaneous ulcerations healed (Fig 3). Investigation of the patient’s underlying immunodeficiency revealed deficiencies in both B- and T-cell function (Table I) and she was given a diagnosis of combined variable immunodeficiency.

**Table I:** Summary of immune studies

Immune study	Ref. range and units	April 2022 (presentation)	March 2021 (1 year ago)
Immunoglobulin M	40-230 mg/dL	13 Low	<25 Low
Immunoglobulin E	0.0-24.9 kU/L	7.9	12.5
Immunoglobulin A	70-400 mg/dL	115	165
Immunoglobulin G	700-1600 mg/dL	633 Low	643 Low
Immunoglobulin GG1	240-1118 mg/dL	342	—
Immunoglobulin GG2	124-549 mg/dL	141	—
Immunoglobulin GG3	21-134 mg/dL	62	—
Immunoglobulin GG4	1-123 mg/dL	15	—
CD3^+^CD4^+^%	28% to 65%	44	52
CD3^+^CD8^+^%	12% to 40%	26	16
CD3%	58% to 87%	71	69
CD3^+^CD4^+^ absolute count	433-1692 /microL	454	151 Low
CD3^+^CD4^+^ absolute count	147-1068 /microL	269	46 Low
CD4/CD8 ratio	0.9-3.6 Ratio	1.7	3.3
Rheumatoid factor	<12.5 IU/mL	<10.0	—
Antinuclear antibody screen	Negative	Negative	—

## Discussion

A recent review of acanthamoeba infections identified that affected individuals often present with system symptoms such as, fever, headache, dizziness, nausea, altered mental status, seizures, and facial palsies prior to cutaneous manifestations.^2^ Disseminated acanthamoebiasis occurs over weeks to months and presages poor prognosis with CNS involvement, inevitably resulting in fatality. For a patient, such as ours, to have cutaneous lesions for a prolonged period of time without further systemic involvement is atypical. In this case, the patient had a concomitant cryptococcal infection and was receiving treatment with long-term outpatient daily fluconazole maintenance therapy. We believe that fluconazole’s activity against free living amoeba, such as Acanthamoeba, prevented dissemination or a more fatal granulomatous encephalitis. In addition to azoles, other medications with proven reported success against acanthamoebiasis include amphotericin, pentamidine, 5-flucytosine, azithromycin, sulfamethoxazole-trimethoprim, miltefosine, and silver nitrate.^3^, ^4^, ^5^

Diagnosing cutaneous acanthamoebiasis is challenging, given its variable clinical presentation and lack of pathognomonic findings.^6^ Lesions frequently occur on the face and extremities and exhibit heterogenous morphology, ranging from papules, pustules, nodules, ulcers, eschars, or abscesses.^7^ Histopathologic diagnosis can be equally difficult, as acanthamoeba cysts and trophozoites resemble large histiocytes.^4^ Several cases of cutaneous acanthamoeba have been reported that were initially misdiagnosed on routine pathology.^8^^,^^9^ The diagnosis is often first made with polymerase chain reaction or culture and later confirmed by identifying cysts and trophozoites with the characteristic “fish eye” nucleus upon reinspection of pathology.

Data on diagnosis and treatment of cutaneous acanthamoebiasis are limited. This case highlights the potential role of dermatological evaluation and the importance of a thorough clinical investigation in immunocompromised patients. Providers should be mindful of potential anchoring bias, especially in the setting of patients with complicated concomitant diseases, such as our patient with a history of cryptococcal infections. In cases where clinical and histopathology diagnosis is difficult, polymerase chain reaction may be warranted and is increasingly utilized in the workup of challenging cases where an infection is suspected. Additionally, prophylactic therapy in immunocompromised patients may alter clinical presentation and ultimately delay further infectious spread. Lastly, we describe the successful use of fluconazole, flucytosine, and miltefosine as treatment for the rare entity of cutaneous acanthamoebiasis lacking CNS involvement.

## Conflicts of interest

None disclosed.
